# Herbalism and glass-based materials in dentistry: review of the current state of the art

**DOI:** 10.1007/s10856-023-06764-w

**Published:** 2023-11-14

**Authors:** Lamia Singer, Christoph Bourauel

**Affiliations:** 1 Oral Technology, University Hospital Bonn, 53111 Bonn, North Rhine-Westphalia Germany; 2 Department of Orthodontics, University Hospital Bonn, 53111 Bonn, North Rhine-Westphalia Germany

## Abstract

Half a million different plant species are occurring worldwide, of which only 1% has been phytochemically considered. Thus, there is great potential for discovering novel bioactive compounds. In dentistry, herbal extracts have been used as antimicrobial agents, analgesics, and intracanal medicaments. Glass-ionomer cement (GIC) and bioactive glass (BAG) are attractive materials in dentistry due to their bioactivity, adhesion, and remineralisation capabilities. Thus, this review summarizes the evidence around the use of phytotherapeutics in dental glass-based materials. This review article covers the structure, properties, and clinical uses of GIC and BAG materials within dentistry, with an emphasis on all the attempts that have been made in the last 20 years to enhance their properties naturally using the wisdom of traditional medicines. An extensive electronic search was performed across four databases to include published articles in the last 20 years and the search was concerned only with the English language publications. Publications that involved the use of plant extracts, and their active compounds for the green synthesis of nanoparticles and the modification of GIC and BAG were included up to May 2023. Plant extracts are a potential and effective candidate for modification of different properties of GIC and BAG, particularly their antimicrobial activities. Moreover, natural plant extracts have shown to be very effective in the green synthesis of metal ion nanoparticles in an ecological, and easy way with the additional advantage of a synergistic effect between metal ions and the phytotherapeutic agents. Medicinal plants are considered an abundant, cheap source of biologically active compounds and many of these phytotherapeutics have been the base for the development of new lead pharmaceuticals. Further research is required to assess the safety and the importance of regulation of phytotherapeutics to expand their use in medicine.

**Graphical Abstract:**

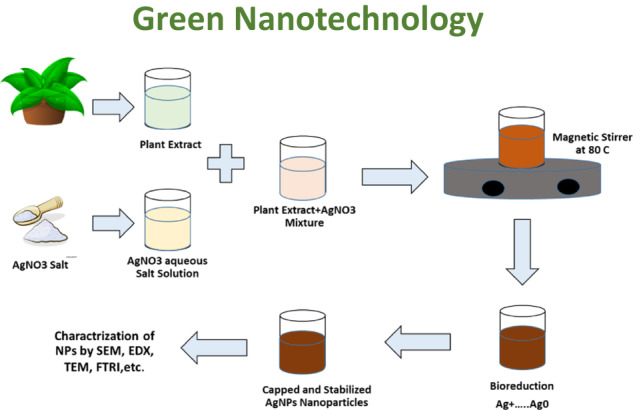

## Introduction

Phytomedicine is a plant-based traditional medical practice that uses various plants, plant parts, or extracts to treat and prevent various health conditions [1]. The global rise in disease incidence, increased antibiotic resistance, opportunistic infections in immunocompromised patients, and financial considerations in developing countries have urged the need to find safe and economical alternatives [2]. Medicinal plants have been used habitually for the cure of numerous health conditions for thousands of years worldwide. In rural areas of developing countries, traditional medicine (TM) is a primary source of remedy, and around 25% of medications are based on plants and their extracts [3, 4]. As per the World Health Organization, 80% of the world’s population relies on herbals for their simple healthcare problems as these plant extracts were accessible, affordable, and culturally acceptable [5].

Traditional medicines (TM) are based on natural products and are of great importance in many cultures. The largest traditional medicinal systems in the world, comprising Chinese medicine, Ayurvedic medicine, Kampo, and Unani medicine in which the use of herbs is the core part of all systems [6, 7]. Traditional Chinese Medicine (TCM) includes thousands of plant species, of which 492 are well-identified and cultivated while the remaining are wild [8]. Traditional Chinese medicine is still common in China in which more than half of the population regularly uses natural preparations, with the highest prevalence of use in rural areas [9, 10]. On the other side, Ayurveda remains one of the most historic traditional medicine systems (4000 BC–1500 BC) that is still widely used in India, Sri Lanka, and other countries and with thousands of registered practitioners in these countries [6]. The word “Ayurveda” literally means knowledge (Veda) of life (Ayu). It aims to preserve health, well-being and prevent disease rather than treat it [11].

Unani medicine is the Western version of Greek and Arabic traditional medicines and it dates back 2500 years. It is based on the accumulated medical knowledge from Egypt, Persia, and Babylon [8]. In the mid-1970s, Unani practices attracted considerable attention worldwide, especially in India, where it has been integrated into the national healthcare system [12]. Kampo is the traditional Japanese medicine that was introduced from China via the Korean peninsula between the fifth and sixth centuries. Kampo herbal formulas have been approved by the Japanese government and have been incorporated into the healthcare system [13].

The desire to capture the wisdom of traditional healing systems led to a rebirth of interest in herbal medicines in Europe and North America at the end of the twentieth century. The increasing interest in self-care improvement has resulted in enormous growth in the popularity of traditional healing modalities, particularly the use of herbal remedies [14]. Although the benefits of TM have been undervalued in many developed countries, herbalism significance in addressing chronic diseases has been emphasized across various communities worldwide [15].

Many studies nowadays have emphasized the important role of TCM, Ayurveda, and Unani systems in the management of oral diseases [16–18]. For example, toothbrushes, made from natural healing plants, are commonly used in many cultures and countries. Moreover, it has been postulated that medicinal plants contain volatile oils, which stimulate blood circulation, tannins that tighten and cleanse gum tissue, and other components, such as vitamin C that preserve gum health [19]. However, due to the absence of a randomized controlled clinical trial, the toxicity of these plant-based medicines is not well known, and up until now, only a few plants have been approved for their worthy medicinal properties [3].

Glass ionomer cement (GIC) and bioactive glass (BAG) are promising vehicles for the transport of many therapeutic agents. In recent years, some work has been done to modify GIC and BAG properties through the incorporation of natural plant extracts and/or their derivatives. Thus, this review article aimed to gather all the recent advances and modifications of both materials and highlight the prospective direction of future research with these versatile bioactive therapeutics.

## Introduction to glass ionomer cement

During the old ages, teeth restorations were made from bone and tusks and later these involved waxes, honey, plant resins, powdered pearl, metals, etc [20]. Amalgam was introduced in the United States in 1833, and afterward, Pierce introduced zinc phosphate cement (1879), and the material ruled in the 19^th^ century together with clove oils and zinc oxide-cement which was very popular in this period [20]. In the 20^th^ century, Smith (1968) introduced polycarboxylate; similarly, Wilson and Kent invented glass ionomer cement (GIC) around this period (1969) and in 1972, GIC was officially introduced to the clinical practice 1072 [21].

Glass-ionomer cement (GIC) is a widely used dental material that is commercially available as hand-mixed and encapsulated versions for clinical use as a restorative material, luting agents, liners, and bases under amalgam restorations [22]. GICs have several attractive properties such as chemical bonding to the tooth structure and fluoride-releasing and recharging abilities [23]. The International Organization for Standardization (ISO) officially designates them as “glass polyalkenoate cement,” yet the term “glass ionomer” is commonly acknowledged as an acceptable informal name and is extensively employed within the dental field [24].

**Fig. 1:**
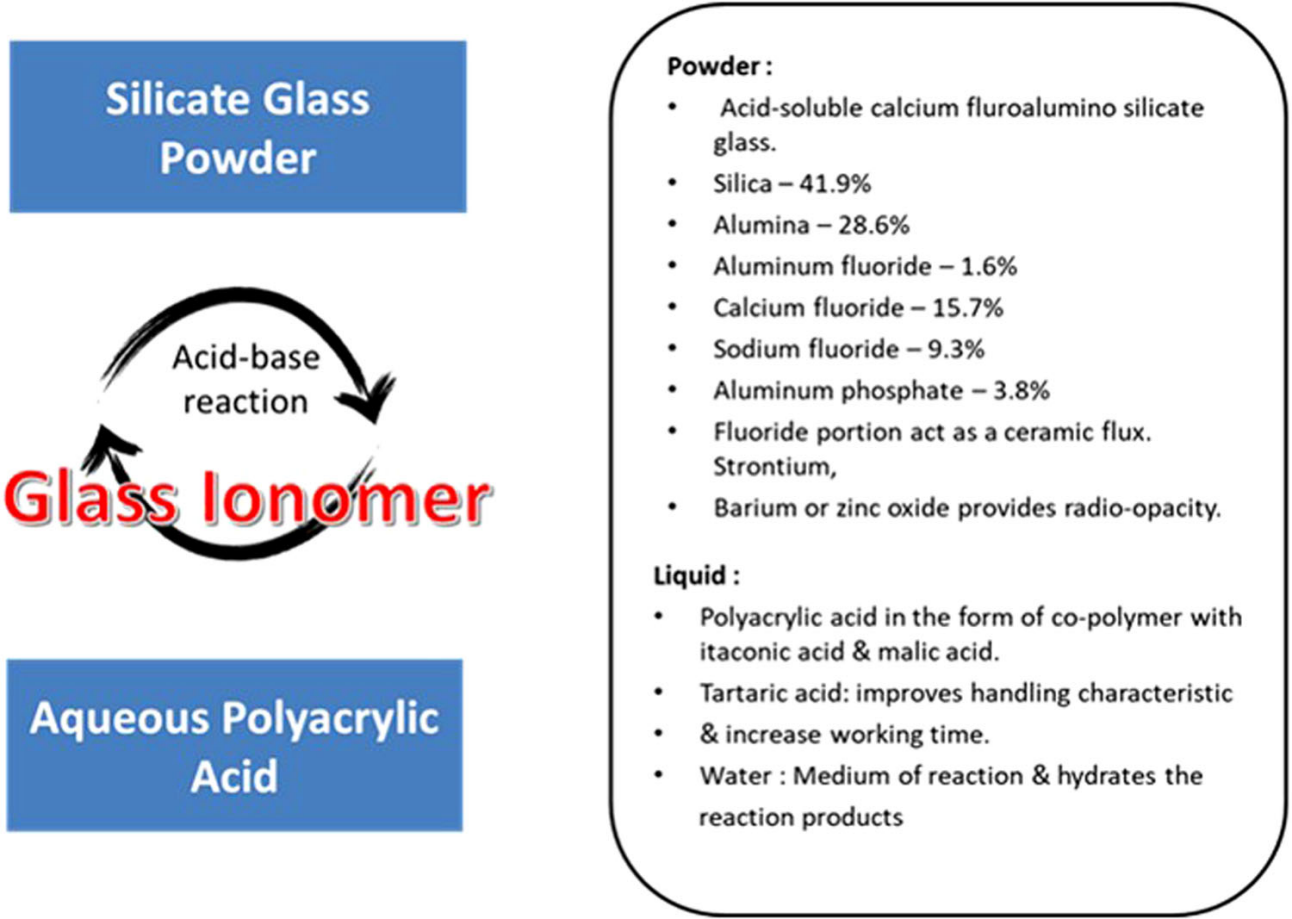
Glass ionomer cement composition

**Fig. 2:**
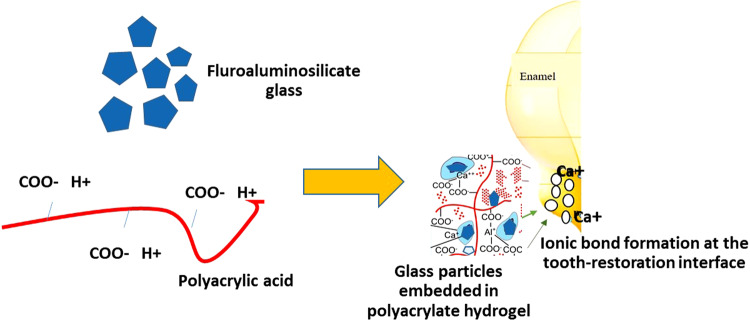
Setting reaction of glass ionomer cement

GICs typically consist of calcium or strontium alumino-fluorosilicate glass and an aqueous polyalkenoic acids, either homopolymer poly(acrylic acid) or the copolymer of acrylic acid and maleic acid [21] (Fig. 1). On mixing the glass and the acid, degradation of the glass occurs, leaching out ions particularly Ca^2+^ (or Sr^2+^) and Al^3+^ ions, into the surrounding medium. This results in an acid-base neutralization reaction, which cross-links acidic polymer chains with multivalent counter ions (Ca^2+^ and Al^3+^) [24]. The setting reaction typically takes 2–5 min after mixing the components of the cement. Moreover, silica and phosphate ions are also released from the glass during the setting reaction to form an inorganic network within the reproduced matrix [25] Fig. 2.

Calcium in the glass may be replaced by strontium, barium, whereas, lanthanum, a rare earth metal is usually added for radiopacity [26]. There are two major problems with the acid part of the glass-ionomer cement. The first problem is the very close attachment of the carboxylic acid groups (COOH) to the backbone of the polymer; which prevents them from complete transformation into carboxylate groups during the setting reaction to form salt bridges [27]. The second problem is related to the molecular weight of the polyacid [28]. Furthermore, it’s noteworthy that most silicate glasses exhibit resistance against acid attack, primarily owing to the strong covalent bonds between silicon (Si) and oxygen (O). Nevertheless, the susceptibility of these glasses to acid attack increases when the ionic properties of silicate materials become more pronounced. Silicate glasses that are vulnerable to acid attacks include aluminosilicate compositions with a notably high ratio of aluminum (Al) to silicon (Si) in which the reactivity and setting time of of the cement are highly dependent on this ratio [27].

## Recent advances and modifications of glass ionomer

Glass ionomer cement has certain characteristics that are attractive for clinical use. They bond adhesively to enamel and dentin, release fluoride ions over a prolonged period, are tooth-biocompatible and have approximately the same coefficient of thermal expansion as that of tooth structure [29]. Despite these advantages, conventional glass ionomers suffer from short working times, long setting times, brittleness, low fracture toughness, poor resistance to wear, and susceptibility to moisture contamination or dehydration during the setting reaction [23].

Various modifications of the powder and liquid of glass ionomer cement have been done over the years to improve its clinical performance. All the modifications that have been done to the powder and liquid of GIC to enhance its clinical performance are listed in Tables 1 and 2.

**Table 1:** Modifications of the liquid of glass ionomer cement

Modification of liquid	Additives	Significance	Publications
1. Resin-modified glass ionomer	2-hydroxyethyl methacrylate (HEMA), and photo initiator (camphorquinone)	Good adhesion to the tooth structure, esthetics, fluoride release, and rapid hardening by visible light	Berzins et al. [30]
2. Oxalic acid	Oxalic acid with its two carboxylate groups (0.5%, 5%, 7%)	For the 0.5%, the degree of cross-linking and polysalt bridge formation increased and subsequently the mechanical properties. Increasing the concentration of oxalic acid to 5 or 7% considerably reduced the working time and initial setting time.	Prentice et al. [31]
3. Phosphoric acid	2% phosphoric acid	2% PO4 3 ions in the phosphoric acid acted as a network former and increased the degree of the cross-linking and compressive strength but the compressive strength drops as the concentration of phosphoric acid increases.	Prentice et al. [32]
4. Poly (vinyl phosphonic acid) (PVPA)	PVPA	Better early moisture resistance, improved compressive strength and flexural strength, decreased solubility, and improved adhesion to tooth structure.	Ellis et al. [33]
5. Amino acid-modified glass ionomer cement:	Proline	Better surface hardness properties, shortened setting times, and significantly enhances mechanical properties.	Ansari et al. [34], Moshaverinia et al. [35]
6. N-Vinylpyrrolidone	Poly (acrylic acid (AA)-co-itaconic acid (IA)-co-N-vinylpyrrolidone)	Significantly lower contact angles and higher adhesion in comparison to commercially available GICs, which led to enhanced bond strength to dentin.	Moshaverinia et al. [36]
7. Hyperbranched polyacrylic acids	Hyperbranched poly (acrylic acid) using a self-condensing vinyl polymerization initiator via atom-transfer radical polymerization (ATRP) technique	Significantly higher mechanical properties.	Zhao et al. [37]
8. Polyelectrolytes with antibacterial properties	1. Poly (quaternary ammonium salt) (PQAS)-polyacid significant antibacterial activity 2. Chlorhexidine-hexametaphosphate nanoparticles	1. Significant antibacterial activity, accompanied by an initial reduction in compressive strength (CS) due to increasing chain length. 2. Slow-release devices for soluble chlorhexidine (CHX) which is a potent antimicrobial agent.	Xie et al. [38], Hook et al. [39]
9. Propolis	Ethanolic (EEP) and aqueous extract of propolis	1. A significant antibacterial and antibiofilm activity against *S. mutans* has been documented. 2. EEP increased the microhardness of GIC without affecting the microleakage. 3. Propolis did not have any antibacterial activity against *S. mutans* and on the other hand, it decreased the flexural and shear bond strength.	Topcuoglu et al. [40], Altunsoy et al. [41], Panahandeh et al. [42]
10. Cetrimide (CT) Cetylpyridinum Chloride (CPC) Benzalkonium Chloride (BC)	1% CT, CPC, and BC	High antibacterial effect against the *S. mutans* and *L. casei* bacteria without seriously deteriorating their surface hardness and fluoride‐releasing properties	Kurt et al. [43]
11. Chlorohexidine	5% chlorhexidine digluconate	Better antibacterial outcomes at 1 month and resulted in higher restoration survival rates than unmodified GIC.	Ratnayake et al. 2022 [44]

**Table 2:** Modifications of the powder of glass ionomer cement

Modification of powder	Additives	Significance	Publications
1. Glass cermet	By sintering the metal and glass powders together, thus strong bonding of the metal to the glass was achieved	Compromised esthetics but improved radio-opacity, and abrasion resistance.	McLean [45]
2. Novel Zn-based GIC system	Germanium dioxide (GeO2), zirconium dioxide (ZrO2), and sodium oxide (Na2O)	Better handling properties without affecting the mechanical properties of the modified GIC.	Dicky et al. [46]
3. Montmorillonite clay	Montmorillonite nano clays were dispersed in poly (acrylic acid)	Remarkable reinforcement effect of nano clays due to their high modulus, high strength, and high aspect ratio.	Fared et al. 2014 [47]
4. Condensable GIC	Higher powder-to-liquid ratio, lower water content, and smaller glass particles	Enhanced flexural strength, high compressive strength, chemical bond, no shrinkage, and coefficient of thermal expansion similar to the tooth.	Kraemer et al. [48]
5. Flowable GIC	Low P:L ratio and possessed increased flow	Improve material’s adaptation to tooth structures and increased bond strength to sound dentin.	Lenzi et al. [49]
6. Giomer (pre-reacted glass ionomer)	Hybridization of GIC and composite by using the pre-reacted glass ionomer technology	Significant fluoride release, fluoride recharge, biocompatibility, smooth surface finish, excellent esthetics, and clinical stability.	Condo et al. [50]
7. Fiber-reinforced glasses	Discontinuous short glass fibers or braided polyethylene fiber ribbons	Short fibers provided effective toughening of the RMGIC matrix by a fiber bridging mechanism. Continuous braided and pre-impregnated fibers had higher flexural strength than discontinuous glass fibers.	Tanaka et al. [51]
8. Reactive fiber-reinforced GIC	Reactive glass fibers of the same composition as the base ionomer	Compression strength for the GIC material was increased from 64 to 170 MPa.	Lohbauer et al. [52]
9. Titanium nano powder	TiO_2_ /NP	Superior mechanical and antibacterial.	Ibrahim et al [53]
10. Bioactive glass	Bioactive glass (BAG)	Bioactivity and tooth regeneration capacity. Biomineralization.	Yli-Urpo [54], Ana et al. [55], Kim et al. [56]
11.Hydroxyapatite	Nano-sized apatite	Better biocompatibility and increased release of fluoride ions.	Moshaverinia [57] Lucas et al. [58]
12. Zirconia containing GIC	Nano-zirconia fillers	Improved mechanical properties and, better esthetics, antimicrobial activity against *Streptococcus mutans*, and sustained high fluoride release.	Chalissery et al. [59]
13. Chitosan GIC	Quaternised chitosan-coated mesoporous silica nanoparticles	Strengthening effect as well as a profound antibacterial effect.	Elshenawy et al. [60]
14. CPP-ACP containing GIC	Casein phosphopeptides (CPP)	Increased compressive strength and micro tensile bond strength and significantly improved release of calcium, phosphate, and fluoride ions at neutral and acidic pH.	Aggarwal et al. [61]
15. Niobium pentoxide	5 wt% Nb_2_O_5_	The physical and chemical properties were not affected but the radiopacity of the GIC was improved.	Garcia et al. [62]
16. Fluorinated graphene	Graphene oxide	Improved mechanical, tribological, antimicrobial properties, and non-altered color properties.	Sun et al. [63]

## Glass ionomer and phytomedicine

Various modifications of GIC with natural plants and their derivatives are listed in Table 3. Green tea is rich in active polyphenols including catechin. Epigallocatechin 3-gallate (EGCG) is the most abundant and potent green tea catechin which has strong antioxidant, anti-inflammatory, antidiabetic, and cancer-preventive properties. The effect of the addition of epigallocatechin-3-gallate on the antibacterial and physical properties of glass ionomer cement (GIC) has been tested. GIC containing 0.1% (w/w) EGCG showed improved mechanical and antibacterial properties with no negative impact on fluoride ion release [64].

**Table 3:** Modifications of GIC with plant extracts and their derivatives

Modification	Significance	Publications
1. Epigallocatechin-3-gallate (EGCG)	EGCG is a promising additive that improved mechanical and antibacterial properties.	Hu et al. [64]
2. *Salvadora persica*	Superior antibacterial properties and clinical performance after 6-month follow-up	Kabil et al. [65]
*3. Disocorea altissima*	Active against *Streptococcus mutans* and has better mechanical properties than the original cement	Kabadayan et al. [66]
*4. Dioscorea altissimo*	Profound photosensitizer for photodynamic therapy (PDT) against *Streptococcus mutans*	Chiode et al. [67]
*5. Salvia officinalis*	*S. officinalis* extract, as an antibacterial agent in GIC was effective against oral pathogens.	Shahriari et al. [69]
6.*Schinusterebinthifolius Raddi* (Brazilian pepper BP)	GIC-BPE has antimicrobial activity at non-cytotoxic concentrations in human fibroblasts cells	Pinto et al. [70]
*7. Salvadora persica, ficus carcia, and Olea europaea* mixture	Plant extracts mixture enhanced the antimicrobial activity of GIC against *Streptococcus mutans*, and improved the compressive strength of GIC.	Singer et al. [71]
8. Proanthocyanidin- grape seed extract (Pa- GSE)	6.5% Pa- GSE could increase the shear bond strength of less resistant material like conventional glass ionomer cement.	Atabek et al. [73]
9. Curcumin and riboflavin	Riboflavin and curcumin have reduced the microleakage within the enamel surface restored with RMGIC.	Alrefeai et al. [74]. Aljamhan et al. [75]
10. Curcumin nanocrystals	1. Non-altered physicochemical properties and good antibacterial results of GIC-containing curcumin nanocrystals were recorded. 2. Curcumin along with O_3_ had the potential to be used as a disinfectant in caries-affected dentin as it improves SBS to RMGIC.	Moghaddam [76] Al Hamdan [77]
11. Cellulose fibers	Discontinuous cellulose microfibers considerably increased the toughening performance of GICs.	Garoushi et al. [78]
12. Gallic acid	GA improved the antibacterial effect against *S. Mutans* and the fluoride release.	El Sharkawy [79]

*Salvadora persica* (*S. persica*, Miswak), is an evergreen shrub, cultivated in India, Pakistan, Amman, and Egypt. Miswak extract possesses different antimicrobial and antifungal properties due to the presence of trimethylamine, chlorides, fluoride, saponins, flavonoids, and phenols. Kabil et al. evaluated the efficacy of adding chlorhexidine gluconate (CHX) or aqueous miswak (*Salvadora persica*) extract on the clinical performance and the antibacterial activity of conventional glass ionomer cement (GIC). CHX and miswak improved significantly the antibacterial properties without seriously affecting the clinical performance of the GIC up to 6 months of follow-up [65].

*Disocorea altissimo* is from the genus *Dioscorea* that is indigenous to Brazil, Bolivia, Peru, Central America, and the Caribbean, *Dioscorea* species reported promising antioxidant, antimicrobial, anti-inflammatory, and thrombolytic activities. Mechanical properties and antimicrobial activity of a glass ionomer cement (GIC) incorporated with an antimicrobial agent from the aerial parts of *Dioscorea altissimo* have been investigated. The modified GIC was very active against *Streptococcus mutans* and had better mechanical properties than the original cement [66].

More recently, *Dioscorea altissimo* (EA) has been further incorporated into GIC as a photosensitizer for Photodynamic therapy (PDT) against *Streptococcus mutans*. PDT has been specified as an adjunct technique to improve antimicrobial activity against dental caries causative microorganisms. EA was found to potentiate the antimicrobial action of GIC against *S. mutans* and therefore, results indicate that EA could be a potential photosensitizer for a PDT [67].

*Salvia officinalis*, commonly known as sage, is a perennial evergreen plant native to the Mediterranean region. Sage extracts exhibit remarkable biological effects due to the presence of polyphenols, monoterpenes, diterpenes, triterpenes, and phenolic components [68]. The antimicrobial activity of GIC modified with *Salvia officinalis* extract powder (0.5%, 0.75%, 1%, and 1.25%) was examined. The study revealed a direct inhibitory activity of sage extract-modified GIC against *S. mutans* and *L. casei* in a dose-dependent manner. Therefore, the authors have claimed that the material can be used as a base, liner, and restorative material in the management of carious teeth [69].

In 2020, a new drug delivery system based on a glass-ionomer-Brazilian pepper (*Schinus terebinthifolius Raddi*) extract combination was assessed to check the activity against pathogenic microorganisms in the oral cavity. The modified material revealed a profound antibacterial activity at non-cytotoxic concentrations for the human fibroblast MRC-5 cells. The results suggested that the modified GIC represents a new low-cost therapeutic option for use in dental treatment, especially in developing countries [70]. In the same year, the antimicrobial properties and compressive strength of GICs modified with a mixture of plant extracts (*Salvadora persica, ficus carcia, and Olea europaea*) at three different concentrations were evaluated. The extracts mixture boosted the antimicrobial activity against *S. mutans* and *M. luteus* while the compressive strength was improved by the addition of the plant extracts mixture at high concentrations [71].

Proanthocyanidins (PA) are polyphenols that are found in many plants, such as cranberry, blueberry, and grape seeds. PA is a natural antioxidant, free-radical scavenger and it induces the formation of exogenous collagen crosslinks [72]. Proanthocyanidin from grape seed extract (Pa-rich GSE) has been shown to enhance the mechanical properties of demineralized dentin. Atabek et al. compared the effect of proanthocyanidin-rich grape seed extract in two different concentrations (6.5% and 12.5%) on the bond strength to dentin for four different cement groups (resin cement, RMGIC, calcium aluminate GIC, GIC). The proanthocyanidin (6.5%) increased the bonding of conventional glass ionomer cement to dentin. While there was no difference in the shear bond strength between resin and calcium aluminate glass ionomer cement at the 12.5% Pa-rich GSE concentration [73].

Only plants and certain microorganisms can synthesize riboflavin and thiamine and the synthesis process is performed by a very complex mechanism. The impact of using curcumin and riboflavin as photosensitizers on microleakage in class V RMGIC restorations was evaluated. The microleakage has been reduced by these photosensitizers without negatively impacting the bond strength. However, it was found that extreme caution should be taken while considering riboflavin and curcumin for class V RMGIC restorations extending to the cementum surface [74, 75].

Curcumin is a polyphenolic agent of the *Curcuma longa L*. plant, which is well known for its numerous biological effects such as anti-inflammatory, antioxidant, and antibacterial activities. An in vitro study was performed to prepare glass ionomer cement (GIC) containing curcumin nanocrystals for the cementation of dental crowns. The modified cement containing curcumin nanocrystals showed a significant increase in antibacterial activity and non-altered physicochemical properties [76]. Moreover, Al-Hamdan assessed the shear bond strength (SBS) of caries-affected dentin bonded to a dental glass ionomer cement after being disinfected with curcumin/O_3_ and chlorhexidine. Curcumin/O_3_ showed improved SBS of RMGIC and has the potential to be used as a disinfectant [77].

Cellulose is a natural product, extracted from abundant sources like plant-based materials and some bacteria. They have desirable characteristics, such as low cost, low density, resistance to compression; rigidity, being non-abrasive, and non-toxic. Discontinuous cellulose microfibers were prepared from birch (Betula species) and aspen (*Populus tremula*) and added to glass ionomer cement at various mass ratios (1, 2, 3, 4, and 5 mass %). The experimental GIC (5% mass) had significantly higher resistance to fracture than the control material. On the other hand, GIC with 1 % mass displayed the highest compressive strength among all tested groups. Thus, it was concluded that the use of discontinuous cellulose microfibers with conventional GIC matrix considerably increased the toughening performance compared with the particulate GICs used [78].

Gallic acid is a secondary polyphenolic compound, extracted from various plants such as oak bark, apple peels, tea, grapes, pineapples, bananas, and Caesalpinia mimosoides plants. Assessment of antibacterial effect, fluoride release, working, and setting time of GIC modified with gallic acid (GA) was performed. Gallic acid improved the antibacterial effect of GIC against *S. Mutans* and the fluoride release. Moreover, although an increase in the working and setting time of GICs was observed, values were still within the limit given by ISO 9917–1:2007 specifications [79].

## Introduction to bioactive glass (BAG)

In the late 1960s and early 1970s, Professor Hench came up with a new bone-biocompatible material using silica (glass) as a base material that could be mixed with other ingredients such as calcium to unite fractured bones [80]. This glass material dissolves in normal physiological conditions, stimulating genes controlling osteogenesis and growth factors (within 48 h) leading to the bone formation of equivalent quality to natural bone [81]. Moreover, after implantation of this material in bone tissue, it resisted removal from the implant site and was described as “bonded to bone and named bioactive glass (BAG). Bioactive glass material is composed of 45% SiO_2_, 24.5% Na_2_O, 24.5% CaO, and 6% P_2_O_5,_which are minerals that occur naturally in the body, and the molecular proportions of the calcium and phosphorus oxides are similar to those in the bones [82].

At the beginning of 1985, bioactive glasses have been proposed as ideal materials for several clinical applications, including middle-ear repair, frontal sinus obliteration, oral-facial applications, and orbital floor reconstruction [83–86]. It has been hypothesized that the dissolution of sodium ions causes an initial increase in the local pH to 8 and then 11 within 8 h [87]. Although BAG alone may have antimicrobial activity as the high alkalinity provides a bactericidal environment, this anti-bacterial activity was observed to decline in vivo due to buffering action of the system [88].

Bioactive glasses have a wide range of applications in dentistry. These glasses can be used as particulates, monolithic shapes and porous or dense constructs in different applications [20]. Bioglass was investigated in many dental applications including implant coatings, bone substitute, scaffolds for bone tissue engineering, regenerative medicine, hypersensitivity and tooth remineralisation [20]. Moreover, the compositional similarity to the bone and tooth structure combined with the bioactive properties and apparent antimicrobial properties inspired researchers to use BAGs as bone substitutes in dentoalveolar and maxillofacial reconstruction, periodontal regeneration, and implants [89].

**Fig. 3:**
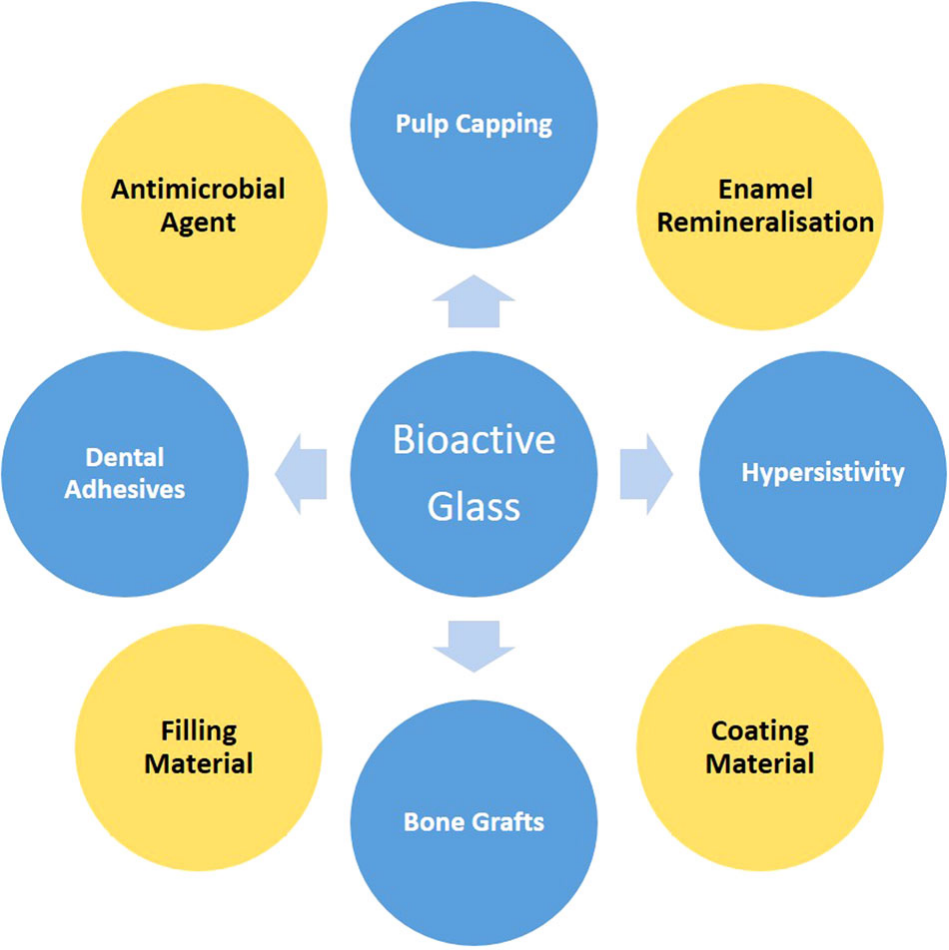
Bioactive glass applications in dentistry

The different applications of BAG in dentistry which have been reported in the last two decades are illustrated in Fig. 3.

## Bioactive glass and traditional medicines

Moreover, BAGs have been used successfully as a substrate to carry growth factors (Bone morphogenic protein), antibiotics, and antimicrobial agents, such as metal oxides (ZnO, MgO, Al2O3, Ag, TiO2) [90–92]. Although metal oxides are widely used to provide antimicrobial properties against a variety of infections, they may cause metal toxicity to humans as in silver upon its accumulation if the release is not well controlled [93]. As a natural expansion of the approach of using BG as a substrate, bioactive glasses were studied as potential candidates for the incorporation of natural herbs and plant extracts aiming to enhance biological performance as well as provide an alternative biomaterial for a variety of applications [89, 94]. Modifications of bioactive glass with phytotherapeutics are listed in Table 4.

**Table 4:** Modifications of bioactive glass with plant extracts and their derivatives

Modifications	Significance	Publications
1. *Yunnan Baiyao*	Accelerated wound healing in diabetic patients.	Mao et al. [95]
2. Gallic acid, Polyphenols from green tea, and red grapes	Ability to induce hydroxyapatite precipitation in SBF.	Cazzola et al. [96]
3. *Epimedium sagitattum*	Enhanced osteogenesis and angiogenesis.	Jing et al. [97]
4. *Salvia officinalis* L.	Bioactive membranes and 3D scaffolds for bone tissue engineering.	Dziadek et al. [98]
5. *Azadirachta indica*	Potent antimicrobial agent for preventing microbial infection.	Prahbu et al. [99]
6. Rice hull ash	Better metabolic activity and viability than that obtained from commercial silica.	Yücel et al. [100]
7. *Ocimum sanctum* and *Curcuma longa*	Halt the leakage of metal and metal oxides, and speed up Osseointegrationtion and antimicrobial effects.	Floroian et al. [104]
8. Curcumin and Chitosan	Formation of apatite-like layer upon immersion in simulated body fluid (SBF).	Virk et al. [105]
9. *Lawsonia inermis*/chitosan	Convenient wettability for the initial protein attachment and apatite crystals formation.	Rehman et al. [106]
10. *Boswellia sacra*	BAG with the therapeutic effects of *Boswellia sacra* represents a novel candidate for the development of tissue healing and regenerative materials.	Ilyas et al. [109]

*Yunnan Baiyao* is a traditional Chinese plant sold and used as an alternative hemostatic agent for humans and animals. In 2014, *Yunnan Baiyao* was used to prepare an ointment by mixing it with 45S5 bioactive glass, which showed accelerated recovery of diabetes-impaired skin wounds [95]. Moreover, in another study, the surface of the bioactive glass was modified with gallic acid and natural polyphenols extracted from red grape skin and green tea leaves in which a fast in vitro bioactivity has been observed [96].

Icariin is a flavonoid compound isolated from a traditional Chinese herb (*Epimedium sagitattum*). It has been used for the treatment of fractures, bone, and joint diseases for hundreds of years. Icariin-loaded-hollow bioactive glass/chitosan therapeutic scaffolds were found to be a promising bone material for enhanced osteogenic differentiation and new bone regeneration [97]. Polymeric and glass-modified composite films were successfully loaded with polyphenols extracted from sage (*Salvia officinalis* L). The modified material exhibited cytocompatibility, and significantly increased expression of bone extracellular matrix proteins (osteocalcin and osteopontin). Furthermore, polyphenol-loaded bioactive glass showed anti-biofilm properties against Gram-positive and Gram-negative bacterial strains [98].

Modification of bioactive glass with several ayurvedic plants such as Neem, rice hulls, Tulsi, turmeric, and *Lawsonia inermis* has been performed by many studies. In 2014, Prabh et al. prepared bioactive glass nanoparticles covered with *Azadirachta indica* (neem) and silver nanoparticles [99]. The antimicrobial tests proved that BG/neem nanoparticles could be used to prevent microbial infection in tissue engineering applications more effectively than BG nanoparticles containing silver. In another study, alkali-extracted silica from rice hull ash was used to prepare bioactive glass with a composition of 6 SiO_2_% –24% Na_2_O ^–^ 24% Ca O ^–^_6_% P_2_O_5_. Results showed that the bioactive glasses from rice hull ash silica had better metabolic activity and viability than that obtained from commercial silica [100].

*Ocimum sanctum* “holy basil” and Turmeric (*Curcuma longa*), with curcumin as the main active compound, are two Ayurvedic medicinal plants that are popular for their active biological properties [101–103]. Floroian et al. coated a stainless-steel implant with thin composites from bioactive glass mixed with antimicrobial turmeric- *Ocimum sanctum* plant extracts. It was concluded that the synthesized layers provided reactivity, antimicrobial effects, and a strong barrier against ion release from the stainless-steel substrate [104].

In 2019, a multilayer coating system based on chitosan/curcumin coatings on poly-ether-ether-ketone/bioactive glass/hexagonal boron nitride (on 316 L stainless steel) was performed by electrophoretic deposition. The multi-coat aimed to produce bioactive and antibacterial coatings for orthopedic implants. The apatite-like layer is similar to hydroxyapatite was formed, confirming the existence of an intact bond between bone and the coated surface [105]. *Lawsonia inermis* (henna), which plays an appreciable role in Ayurvedic medicine, was deposited in combination with chitosan and bioactive glass on top of a polyether ether ketone-coated stainless-steel substrate. The presence of lawsone in the top layer provided an antibacterial effect and induced the formation of apatite-like crystals, indicating the possibility of achieving close interaction between the coating surface and bone [106].

Among the plants that play a very significant role in all of the Unani, Ayurveda, and Chinese medicines is frankincense. Frankincense is an oleo gum resin obtained through a deep longitudinal incision in the trunk of the genus Boswellia trees [107]. Frankincense belongs to the Arabian Peninsula for more than 6000 years and was reintroduced to Europe and traded as far as China. In Ayurveda medicine, frankincense (salai guggul) has been used as an anti-inflammatory, antibacterial, anti-arthritic, and analgesic agent for the treatment of related diseases. In Traditional Chinese Medicine (TCM), frankincense is commonly used as a remedy for improving blood circulation and relieving pain [108]. Ilyas et al. prepared novel amino-mesoporous bioactive glass nanoparticles (MBGNs) loaded with the *Boswellia sacra* alcoholic extract. The modified BAG nanoparticles had high loading volume than the unmodified MBGNs. Moreover, *Boswellia sacra* molecules were released via a controlled diffusion manner and it showed a profound antibacterial effect against *S. aureus* bacteria. Moreover, results of cell cytocompatibility studies using human osteoblastic-like cells indicated better cell viability of the *Boswellia sacra*-loaded MBGNs as compared to the unloaded MBGNs [109].

## Green nanotechnology

**Fig. 4:**
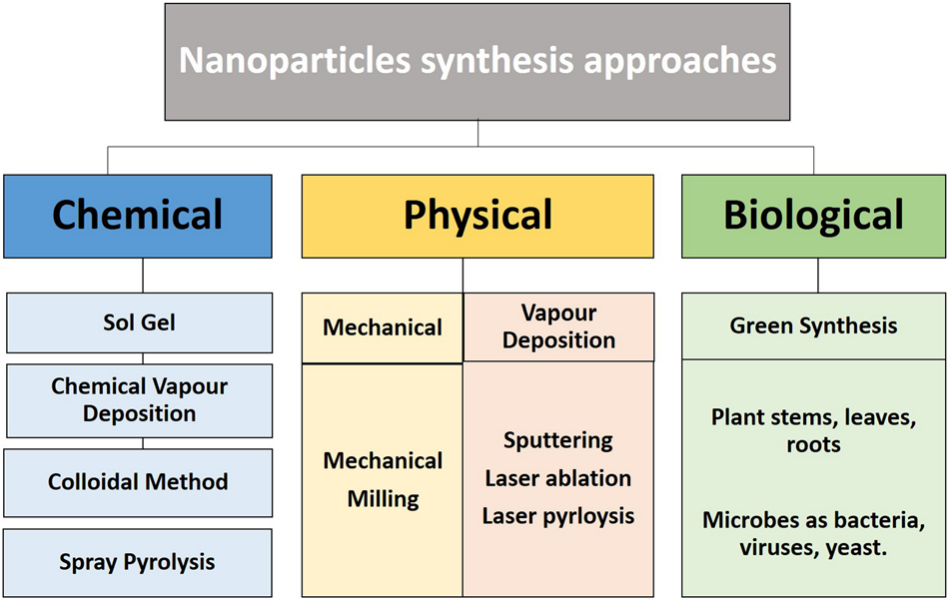
Different methods for synthesis of nanoscale particles

Nanotechnology is an interdisciplinary field that evolved greatly in the twenty-first century. It involves the development, handling, and use of materials in the size range between 1 and 100 nm [110]. Advancements in nanotechnology over the past years have allowed the synthesis of a wide class of materials with many potential applications in the environmental and biomedical fields [111]. There are two main techniques for the production of nanoparticles, which are the bottom-up (chemical) and top-down approaches (physical) [112]. Additionally, and more recently, the biological method was introduced, which is a bottom-up approach that synthesizes nanoparticles using viruses, algae, bacteria, yeasts, plants, and fungi [113]. Nanoparticle synthesis via the biological and physicochemical approaches is illustrated in Fig. 4.

**Fig. 5:**
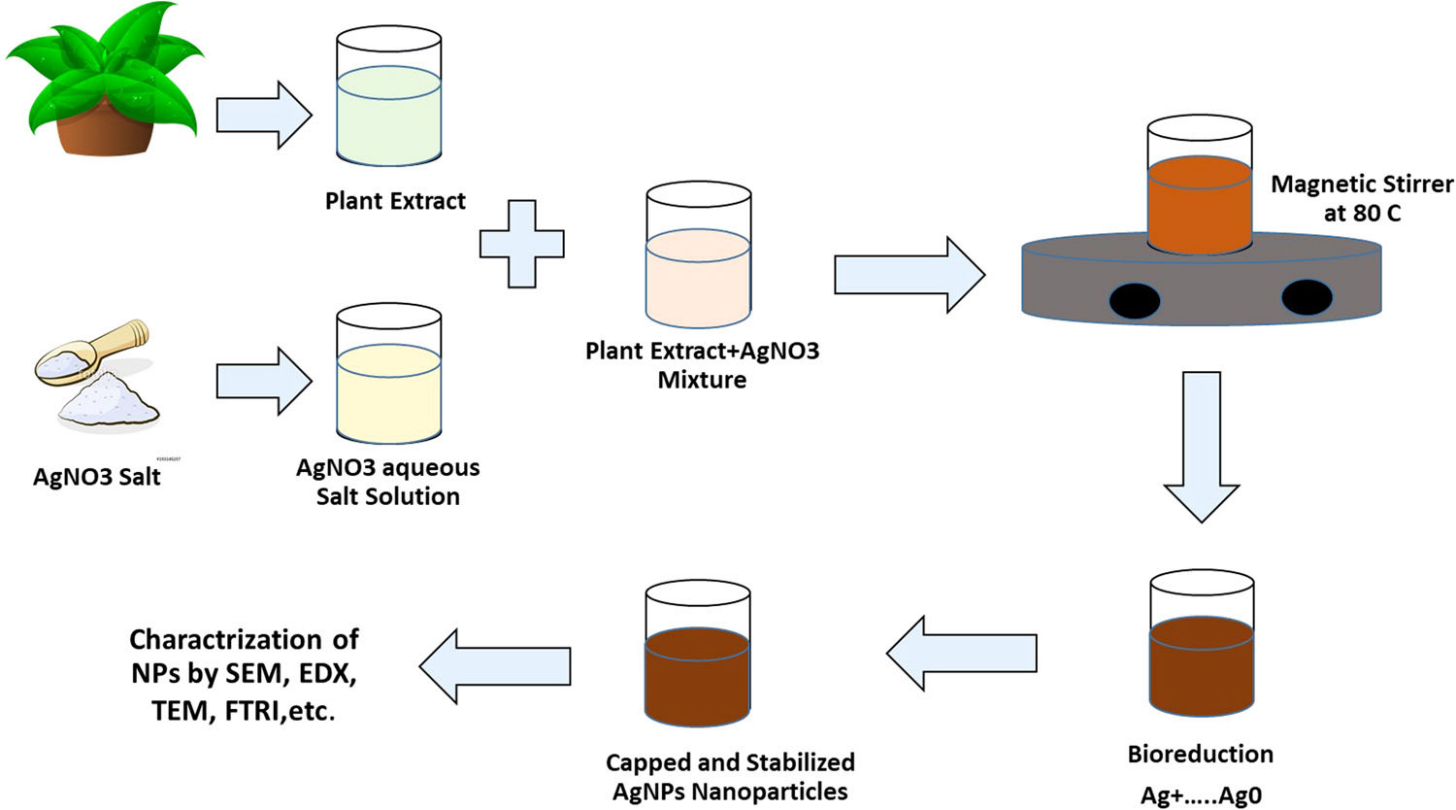
Green synthesis of metal ion nanoparticles using a natural plant extract

Plants and their extracts are considered cheap, safe, abundant, and easy-to-use sources for the synthesis of various types of nanoparticles. The green synthesis process utilizes the aqueous or alcoholic extracts of the plant that are rich in various phytochemical compounds (e.g., phenols, amino acids, flavonoids, alkaloids, saponins, terpenoids, and tannins). These active compounds act as a reducer, stabilizers, and capping agents in the synthesis and stabilization of nanoparticles from metallic salt solutions [114], Fig. 5. Moreover, the green process relies mainly on several aspects of the type of plant extract, the method of extraction, the volume ratio of the metallic salt solutions to the extract, and reaction conditions (pH, temperature, and incubation time) [114].

Ginger (*Zingiber officinale* Roscoe), is a widely used spice that has been used as a remedy for centuries in Ayurvedic, Unani, and Chinese medicines. Ginger is rich in phytochemical compounds particularly gingerols and shogaols phenolic compounds that are well known for their diverse bioactivity against different resistant bacteria [115]. Zingiber officinale (ginger) was used for the green synthesis of silver nanoparticles. The antimicrobial efficacy and compressive strength of GIC combined with ginger-AgNPs, lyophilized miswak, and chlorhexidine diacetate (CHX) were tested. The combination of GIC with green synthesized AgNPs and chlorhexidine together has shown enhanced antimicrobial efficacy and compressive strength compared to the other combinations alone [116].

Cypress trees have been widely used in folk medicine due to their reported antibacterial, wound-healing, and anti-inflammatory activities. The green synthesis of AgNPs was performed using *Cupressus macrocarpa* extract and the biosynthesized particles were incorporated with amoxicillin into GIC to synergize its effect against *Streptococcus mutans* and *Staphylococcus aureus*. The combination was found effective against both tested strains without impairing the mechanical properties of the cement [117].

Cellulose is one of the most abundant biopolymers in the biosphere, which can be obtained from a broad range of plants and animals. Cellulose and its derivatives have been considered as a template for the synthesis of bio-inspired bioactive glass nanoparticles in environmentally friendly conditions. Pure cellulose and amine-grafted cellulose resulted in nanoparticulate composite formation. Methylcellulose has shown to be an excellent candidate for the synthesis of bioactive glass nanoparticles (55 nm). Moreover, superior bioactivity and mechanical stiffness were observed and accounted for the nature of the cellulose template used for the synthesis of nanoparticles [118].

## Safety concerns

The fact that something is natural does not inevitably make it 100% safe. The common belief of most of the population is that herbs in general are nontoxic but it should be highlighted that those plants and herbal preparations can cause adverse effects, serious allergic reactions, adverse drug interactions, and can interfere with some laboratory tests. The active ingredients of plant extracts are chemicals that are similar to those in purified medications, and they have the same potential to cause serious side effects. In many situations and for many plants the potential toxicity of herbs and their derivatives has not been recognized [119].

Plants and herbs can be generally classified into three major classes with regard to toxicity. The food herbs class such as peppermint, ginger, garlic, lemon, and onions. These herbs have low toxicity and can be consumed without any adverse response (acute or chronic toxicity) over long periods. However, they may elicit an allergic response in certain individuals. The second category is medicinal herbs which need to be used with greater knowledge (certain dosage) for specific conditions (after a medical diagnosis) and usually only for a limited period. They have a greater potential for adverse reactions and drug interactions in some cases. They include *Aloe vera*, black cohosh, comfrey, *Echinacea*, ephedra, *Ginkgo biloba*, *Ginseng*, kava-kava, milk thistle, and *Senna* [119].

The last group compromises the poisonous herbs that have a strong potential for acute or chronic toxicity and should only be prescribed by professionals who understand their toxicology and appropriate use. Fortunately, the vast majority of these herbs are not available to the public and are not sold in health food or herbal stores. *Aconite*, *Arnica* spp., *Atropa belladonna*, *Digitalis*, *Datura*, male fern, *Gelsemium*, and *Veratrum* are some examples of toxic herbs [119].

## Conclusions

In the era of intensive exploration of new natural alternatives to replace synthetic chemicals in dentistry and medicine, herbs and plant extracts have shown to be good candidates for such an aim. Plant extracts and their derivatives have shown to be very successful additives to GIC and BAG for the prevention and treatment of different oral conditions. Herbs have been used as analgesics, anti-inflammatory, and antioxidant drugs and seem to be a feasible alternative to contemporary antimicrobial agents. Moreover, plant extracts have a proven role in the reduction and stabilization of metal ions nanoparticles safely and ecologically. More investigation is needed to prove that herbal medicine is a valid and safe therapeutic strategy in dentistry.

## Data Availability

The datasets used and/or analyzed during the current study are available from the corresponding author on reasonable request.
